# Diversity in human fertility in India beyond age 30: an index based temporal study

**DOI:** 10.3389/fgwh.2026.1882364

**Published:** 2026-07-22

**Authors:** Brijesh P. Singh, Ravi Kant Maurya, Anuj Singh

**Affiliations:** 1 Department of Statistics, Institute of Science, Banaras Hindu University, Varanasi, India; 2 Department of Statistics, Mahatma Gandhi Kashi Vidyapith, Varanasi, India; 3 Department of Sciences (Mathematics), Indian Institute of Information Technology (IIIT), Ranchi, Jharkhand, India

**Keywords:** age-specific fertility rate, diversity index, fertility beyond age 30, India, NFHS, rural-urban differentials

## Abstract

**Introduction:**

Fertility patterns among women aged 30 years and above have undergone substantial changes in India, reflecting the country's ongoing demographic transition. However, evidence on the diversity and distribution of age-specific fertility across states and urban-rural settings remains limited.

**Method:**

The study examines the diversity-index using age-specific fertility rates (ASFR) among women aged 30–49 years across 16 major Indian states and the Union Territory of Delhi, with particular focus on urban-rural differentials over five rounds of the National Family Health Survey (NFHS) from 1992–93 to 2019–21.

**Results:**

Regional and temporal variations in fertility diversity were evident across India. Southern and western states indicates the lower diversity indices, representing more concentrated fertility patterns, whereas northern and eastern states, particularly Bihar, Uttar Pradesh, and Rajasthan, showed greater fertility heterogeneity. Urban areas consistently demonstrated lower fertility diversity than rural areas. Overall, most states experienced a decline in fertility diversity over time, reflecting increasing concentration of childbearing within specific age groups.

**Discussion:**

The findings indicate that fertility beyond age 30 is becoming increasingly concentrated across India, although substantial regional and rural–urban disparities persist. These results highlight the need for region-specific reproductive health and family planning strategies and provide valuable evidence for understanding fertility transitions and informing demographic and women's reproductive health policies.

## Introduction

India, home to over 1.4 billion population, is characterized by remarkable demographic, cultural, and socio-economic diversity. This heterogeneity is reflected not only in language, religion, and cultural practices, but also in patterns of fertility behavior across states and regions. Over the past several years, the country has witnessed a significant decline in fertility levels, a trend closely associated with its demographic transition (1). However, this national trend masks substantial variations across states, districts, and urban-rural divisions which persist in some cases, have even intensified in recent years. Understanding these variations is important for developing targeted reproductive health programs and for achieving population stabilization goals under the National Population Policy (2).

A key metric for studying the structure and patterns of fertility is the Age-Specific Fertility Rate (ASFR), which measures the number of live births per 1,000 women in a specific age group (15–49 years age-group). And total fertility rate (TFR) provides a summary measure of fertility, ASFR to identify the specific ages at which fertility is concentrated or spread. A more comprehensive approach for analyzing ASFR is to assess its diversity or dispersion across age groups, which can be quantified using the Diversity Index. This index captures the extent to which fertility is evenly or unevenly distributed among various reproductive age groups, and it is particularly useful for comparing across geographies over time (3).

The Diversity Index of ASFR serves as an insightful statistical tool to measure fertility heterogeneity within populations. A higher diversity index indicates greater heterogeneity in fertility behavior—implying that fertility is distributed across a wider range of age groups—while a lower value signifies concentration of fertility in fewer age intervals, often reflecting more structured or delayed reproductive behavior. Therefore, this index helps in understanding how fertility transitions are evolving within different age-groups of the population. Over the years, researchers have increasingly used the diversity index to evaluate demographic transitions, examine socio-economic determinants of fertility, and to identify policy priorities in population and health programs.

Moreover, the findings from the National Family Health Survey (NFHS) found the decline trends in ASFR across Indian states from NFHS-1 to NFHS-4 (4, 5). The study reported a modest but notable decline in the diversity index from 0.221 to 0.205 over this period, suggesting an overall movement toward more uniform fertility patterns—largely a result of improved access to education, contraception, and reproductive health services.

Author also investigate the spatial dimensions of fertility across districts and states. Their findings emphasized contrasts between more developed southern states—such as Kerala and Tamil Nadu, which showed low diversity indices indicating concentrated fertility in specific age groups—and states like Bihar and Uttar Pradesh, where high diversity indices signaled prolonged or delayed fertility behavior. These patterns closely correlate with levels of female education, early marriage prevalence, and access to family planning resources (6).

Educated women tend to have children later, and often limit childbearing to fewer age intervals, reflecting informed choices and enhanced autonomy in reproductive decisions (7). This finding aligns with global demographic research showing that education influences fertility behavior by altering aspirations, increasing labor market participation, and expanding knowledge and use of contraceptives. Capturing these disparities is critical, as national-level statistics can obscure the distinct needs of rural populations, where reproductive health services may be limited and cultural norms often support early and repeated childbearing (8).

Therefore, the study has some important objectives like:

(i) to examine temporal changes in fertility diversity among women aged 30–49 years across major Indian states;
(ii) to identify states and regions exhibiting high and low fertility diversity;
(iii) to assess urban-rural differentials in fertility diversity; and
(iv) to understand the implications of these patterns for fertility transition and reproductive health policy in India.

## Data & methods

### Data-source

This study used nationally representative data from the National Family Health Survey (NFHS), one of India's most comprehensive sources of demographic and health-related information. It is conducted by the International Institute for Population Sciences (IIPS), Mumbai, under the stewardship of the Ministry of Health and Family Welfare, Government of India. These surveys provide valuable data on a wide range of indicators related to population, health, nutrition, family welfare, and socio-economic conditions, both at the national and sub-national levels. The analysis used five rounds of NFHS data: NFHS-1 (4), NFHS-2 (9), NFHS-3 (10), NFHS-4 (5) and NFHS-5 (11). This study focuses on sixteenth major Indian states and one Union Territory (Delhi). The selected states represent a diverse mix of regions across India, offering insights into regional fertility patterns and demographic diversity. The classification includes:

- **North and Central States**: Haryana, Punjab, Himachal Pradesh, and Delhi
- **EAG States**: Uttar Pradesh, Madhya Pradesh, Odisha, Bihar, and Rajasthan
- **Southern States**: Tamil Nadu, Karnataka, Kerala, Andhra Pradesh
- **Western States**: Gujarat, Maharashtra
- **Eastern and Northeastern States**: West Bengal, Assam

### Methodology

The diversity index for fertility is calculated using the same formula as the Shannon–Wiener diversity index used in ecology (12). Since this index is calculated from fertility contributions within the age groups of women's 30–34, 35–39, 40–44, and 45–49 years, the study assesses the heterogeneity of fertility behavior in later reproductive ages. It takes into account the proportion of total fertility contributed by each subgroup, and higher values indicate greater heterogeneity in fertility rates across subgroups, while lower values indicate more uniformity. Understanding patterns of fertility across different subgroups is important for designing effective reproductive health policies and programs, as well as for identifying disparities in reproductive health outcomes.

The Diversity index used is given by:

$$DI=\frac{-\sum_{i=1}^{n}\;p_{i}*\ln\;(p_{i})}{\ln\;(n)}*100$$

Where $p_{i}$ is proportion of fertility contributed by the $i^{th}$ age group and *n* is the number of age groups having fertility more than zero.

A higher DI value (closer to 100%) indicates greater diversity in the distribution of fertility across age groups. A lower DI value indicates concentration of fertility in fewer age groups. Understanding and addressing fertility diversity is crucial for achieving sustainable population growth, reducing inequalities, and ensuring reproductive rights for all subgroups.

## Results

Table 1 shows the state-wise diversity index of age-specific fertility rates across five rounds of the National Family Health Survey (NFHS). Overall, most states exhibited a decline in fertility diversity over time, but the pace and magnitude of change varied considerably across regions. Delhi showed a steady decline in DI from 55.05 in NFHS-1 to 44.16 in NFHS-5, indicating increasing concentration of fertility within fewer age groups. Similar declines were observed in Punjab (57.15–46.73), Himachal Pradesh (65.41–35.75), Kerala (68.25–42.75), and Tamil Nadu (55.06–38.94), suggesting a shift toward more structured fertility schedules. Northern and central states recorded higher diversity levels throughout the study period. Uttar Pradesh maintained one of the highest diversity indices, although it declined from 77.42 in NFHS-1 to 57.04 in NFHS-5. Madhya Pradesh and Bihar also exhibited high fertility diversity, with Bihar recording the highest DI in NFHS-5 (60.04). Rajasthan remained among the high-diversity states despite a substantial decline from 74.31 in NFHS-1 to 51.61 in NFHS-5. In contrast, southern states such as Kerala, Karnataka, Andhra Pradesh, and Tamil Nadu experienced notable reductions in fertility diversity, reflecting more concentrated fertility behavior and advanced fertility transition.

**Table 1:** State-wise diversity index in age-specific fertility rates across NFHS rounds (NFHS-1 to NFHS-5).

States	NFHS-1	NFHS-2	NFHS-3	NFHS-4	NFHS-5
Delhi	55.05	48.53	41.21	54.83	44.16
Haryana	69.49	71.07	46.74	65.73	49.22
Himachal Pradesh	65.41	58.85	37.58	53.54	35.75
Punjab	57.15	46.86	45.01	48.63	46.73
Rajasthan	74.31	78.14	66.74	67.28	51.61
Madhya Pradesh	75.95	72.47	74.43	58.51	54.43
Uttar Pradesh	77.42	70.63	69.46	64.97	57.04
Bihar	71.26	67.23	74.56	69.67	60.04
Odisha	57.60	54.27	62.10	55.29	49.68
West Bengal	68.96	64.53	66.77	42.17	49.57
Assam	65.10	58.90	60.43	58.50	58.03
Gujarat	60.33	65.65	47.35	53.80	48.15
Maharashtra	52.48	41.40	46.70	46.42	47.56
Andhra Pradesh	58.62	58.58	62.03	33.32	48.31
Kerala	68.25	56.15	40.00	47.92	42.75
Karnataka	60.57	55.46	46.46	44.12	49.33
Tamil Nadu	55.06	54.62	42.27	37.24	38.94

Table 2 presents the state-wise ranking of the diversity index across NFHS rounds. Uttar Pradesh, Madhya Pradesh, Bihar, and Rajasthan consistently occupied the top ranks during the earlier survey rounds, indicating sustained heterogeneity in fertility timing. Bihar emerged as the highest-ranked state in NFHS-3, NFHS-4, and NFHS-5, highlighting persistent fertility diversity despite the overall fertility decline observed nationally. Uttar Pradesh remained among the top three states throughout the study period, while Madhya Pradesh consistently ranked within the top five. In contrast, states such as Maharashtra, Kerala, Himachal Pradesh, Punjab, and Tamil Nadu generally occupied lower ranks, reflecting more concentrated fertility patterns. Tamil Nadu remained among the lowest-ranked states across all survey rounds and ranked 16th in NFHS-5. Himachal Pradesh experienced one of the most notable changes, declining from Rank 7 in NFHS-1 to Rank 17 in NFHS-5, suggesting increasing concentration of fertility within narrower age intervals. While several states demonstrated substantial shifts over time. Assam improved from Rank 9 in NFHS-1 to Rank 2 in NFHS-5, indicating relatively greater fertility dispersion in recent years. Odisha also improved from Rank 13 in NFHS-1 to Rank 6 in NFHS-5. Conversely, Andhra Pradesh moved from Rank 7 in NFHS-3 to Rank 17 in NFHS-4 before recovering to Rank 10 in NFHS-5.

**Table 2:** State-wise ranking of the diversity index in age-specific fertility rates across NFHS rounds (NFHS-1 to NFHS-5).

States	NFHS-1	NFHS-2	NFHS-3	NFHS-4	NFHS-5
Delhi	16	16	15	8	14
Haryana	5	3	10	4	9
Himachal Pradesh	7	9	17	10	17
Punjab	14	15	13	11	13
Rajasthan	3	1	5	2	5
Madhya Pradesh	2	2	2	5	4
Uttar Pradesh	1	4	3	3	3
Bihar	4	5	1	1	1
Odisha	13	14	6	7	6
West Bengal	6	7	4	15	7
Assam	9	8	8	6	2
Gujarat	11	6	9	9	11
Maharashtra	17	17	11	13	12
Andhra Pradesh	12	10	7	17	10
Kerala	8	11	16	14	15
Karnataka	10	12	12	12	8
Tamil Nadu	15	13	14	16	16

Table 3 shows the diversity index of age-specific fertility rates across five NFHS rounds (NFHS-1 to NFHS-5) separately for urban and rural areas in various Indian states. States like Haryana and Rajasthan reflect relatively high and persistent Diversity Index values in rural areas across all rounds. For example, Haryana's rural index remained high from 52.82 in NFHS-1 to 46.36 in NFHS-5, indicating continued diversity in rural fertility behavior. In contrast, the urban index in Haryana fluctuated more, ranging from 38.11 to 56.04. Himachal Pradesh shows a noticeable contrast, with a significant decline in the urban Diversity Index by NFHS-5 (19.76), while rural values remained relatively stable around the 30s and 60s. In states like Bihar and Uttar Pradesh, both urban and rural areas show high diversity values in NFHS-5 (urban: 52.50 and 53.99; rural: 61.02 and 58.19, respectively), reflecting wide fertility distribution across age groups, especially in rural settings. Kerala and Tamil Nadu report generally lower Diversity Index values, especially in rural areas by NFHS-5 (46.97 for Kerala and 34.33 for Tamil Nadu), suggesting more concentrated fertility patterns possibly influenced by lower fertility rates and effective family planning.

**Table 3:** Diversity index of age-specific fertility rates in urban and rural areas across NFHS rounds (NFHS-1 to NFHS-5).

States	Urban					Rural				
	NFHS-1	NFHS-2	NFHS-3	NFHS-4	NFHS-5	NFHS-1	NFHS-2	NFHS-3	NFHS-4	NFHS-5
Delhi	24.79	29.58	39.58	57.07	46.19	39.26	^a^	40.43	^a^	44.61
Haryana	49.49	42.51	56.04	38.11	50.92	52.82	57.99	73.88	69.97	46.36
Himachal Pradesh	36.47	45.23	38.21	26.95	9.76	34.20	36.28	27.17	65.09	38.95
Punjab	27.17	19.10	35.65	36.32	45.94	55.65	35.32	42.26	27.17	47.61
Rajasthan	34.46	54.87	41.31	46.43	47.89	59.39	55.67	62.03	65.91	54.99
Madhya Pradesh	65.05	48.39	65.94	35.77	53.77	65.56	57.57	58.51	60.33	54.58
Uttar Pradesh	61.35	55.38	59.11	52.96	53.99	61.14	38.00	54.75	53.67	58.19
Bihar	34.50	25.11	78.23	52.17	52.50	51.22	45.50	52.49	59.28	61.02
Odisha	49.93	51.26	45.80	52.38	49.55	37.76	37.02	57.57	25.16	50.30
West Bengal	44.32	19.10	61.82	20.91	51.66	57.54	50.63	56.74	25.16	48.27
Assam	36.91	23.72	37.89	69.36	56.34	43.27	40.42	33.84	59.14	57.80
Gujarat	73.03	32.89	42.53	41.54	42.43	52.95	57.13	32.50	44.20	53.127
Maharashtra	33.61	24.62	49.84	52.87	49.56	49.90	17.29	29.94	27.17	44.40
Andhra Pradesh	28.73	23.72	41.28	26.04	35.55	36.09	33.77	43.15	40.79	53.41
Kerala	34.81	26.40	33.61	52.09	50.81	53.38	34.8	19.56	21.96	46.97
Karnataka	59.05	46.60	53.40	45.52	49.46	54.04	33.72	20.69	52.04	40.11
Tamil Nadu	35.12	47.62	45.94	34.94	42.65	32.50	26.28	35.73	38.62	34.33

a Rate not shown; based on fewer than 125 woman-years of exposure.

Table 4 compares the state-wise rankings of the diversity index in age-specific fertility rates for urban and rural areas across five NFHS rounds and reveals clear rural–urban contrasts. Northern and central states consistently appear in the top rankings, especially in rural areas. Uttar Pradesh ranks highly across all rural rounds (Rank 2 in NFHS-1, Rank 6 in NFHS-3, and Rank 2 in NFHS-5), while Madhya Pradesh also remains consistently strong (Ranks 1–5 across NFHS-1 to NFHS-5). Bihar shows a similar pattern, attaining Rank 1 in rural NFHS-5 and top-10 positions across earlier rounds. Rajasthan maintains high rural rankings as well, including Rank 2 in NFHS-3 and Rank 4 in NFHS-5, indicating persistent fertility diversity in rural northern regions. Urban patterns differ substantially. Assam ranks Rank 1 in NFHS-4 and NFHS-5 (urban), showing very high diversity in urban fertility timing. Delhi performs strongly in selected rounds (Rank 3 in NFHS-4 urban), while states like Gujarat and Karnataka show mid-tier urban positions. In contrast, southern and western states generally rank low. Tamil Nadu, for example, remains at the bottom in almost all rural rounds (Ranks 18, 17, 11, 11, 17) and consistently low in urban rankings (Ranks 10–15). Kerala also shows low diversity in both settings, particularly rural (Rank 17 in NFHS-3 and Rank 16 in NFHS-4). Several states demonstrate rural–urban divergence. Bihar and West Bengal rank high in rural NFHS-5 (Rank 1 and Rank 9 respectively) but much lower in urban (Ranks 4 and 6). Conversely, Assam and Delhi show strong urban patterns but relatively weaker rural rankings. States such as Himachal Pradesh and Maharashtra show consistently low or mid-range rankings in both settings, reflecting more uniform fertility timing.

**Table 4:** Comparative state-wise ranking of diversity index in age-specific fertility rates across NFHS rounds for urban and rural areas.

States	Urban					Rural				
	NFHS-1	NFHS-2	NFHS-3	NFHS-4	NFHS-5	NFHS-1	NFHS-2	NFHS-3	NFHS-4	NFHS-5
Delhi	17	10	13	3	12	15	^a^	10	^a^	13
Haryana	6	8	5	14	7	7	1	1	1	12
Himachal Pradesh	9	7	14	15	17	16	12	15	3	16
Punjab	16	16	16	13	13	5	13	9	12	10
Rajasthan	13	2	11	9	11	3	4	2	2	4
Madhya Pradesh	2	4	2	16	3	1	2	3	4	5
Uttar Pradesh	3	1	4	5	2	2	11	6	7	2
Bihar	12	12	1	7	4	8	7	7	5	1
Odisha	5	3	9	6	9	14	14	4	14	8
West Bengal	7	16	3	17	6	4	5	5	15	9
Assam	8	14	15	1	1	12	10	12	6	3
Gujarat	1	9	10	11	14	6	3	13	9	7
Maharashtra	14	13	7	4	8	9	16	14	13	14
Andhra Pradesh	15	14	12	16	16	17	15	8	10	6
Kerala	11	11	17	8	5	6	14	17	16	11
Karnataka	4	6	6	10	10	5	16	16	8	15
Tamil Nadu	10	5	8	12	15	18	17	11	11	17

a Rate not shown; based on fewer than 125 woman-years of exposure.

## Discussion

As we know diversity index used in this study to measures the dispersion of fertility across reproductive age groups of women and should not be interpreted as a direct measure of fertility magnitude. A population may exhibit a low fertility level while maintaining a relatively high diversity index if births are distributed across a broader range of reproductive ages, particularly at later ages. Conversely, a high fertility level may be associated with a lower diversity index when childbearing is concentrated within a limited number of age groups. Therefore, the Diversity Index provides information on the timing and distribution of fertility rather than its overall quantum (13). This distinction is particularly relevant in populations undergoing fertility transition, where declines in fertility levels may coexist with shifts in the age pattern of childbearing (14). Consequently, the Diversity Index should be interpreted as complementary to conventional fertility indicators such as Total Fertility Rate (TFR) and Age-Specific Fertility Rates (ASFR), offering additional insights into the heterogeneity of reproductive behavior across populations.

Our results show considerable variation in the diversity of ASFR across India's states, with states in the southern and western regions, such as Kerala, Tamil Nadu, and Maharashtra, demonstrating lower diversity indices. These states have long been recognized for their achievements in reducing fertility rates through successful family planning programs, high levels of female education, and better healthcare infrastructure (15). Kerala, in particular, stands out with a fertility transition that began early and has been driven by progressive social policies, which is reflected in its consistently low diversity index across all NFHS rounds. Tamil Nadu and Maharashtra also show significant improvements in fertility rates, likely due to the combined effect of economic growth, female empowerment, and effective family planning interventions (16, 17). In contrast, states in the northern and eastern regions, including Uttar Pradesh, Bihar, and Odisha, exhibit higher diversity indices. These regions have historically lagged behind in terms of socio-economic development and access to quality healthcare. The higher diversity in fertility rates in these states is indicative of the broader challenges they face in promoting reproductive health. The heterogeneity in fertility rates in these states may be a result of disparities in education, income levels, cultural norms, and access to healthcare services. Studies have shown that fertility rates are more heterogeneous in regions where there are significant gaps in gender equality, education, and healthcare access (18). This pattern aligns with the findings of Bhattacharya who noted that fertility diversity is higher in states with less access to family planning services and lower levels of female education (19). These regional disparities emphasize the need for more focused interventions in northern and eastern states, where fertility rates remain high and diverse, often influenced by socio-cultural factors and a lack of access to reproductive health services (20).

Our analysis also demonstrates a significant gap in fertility diversity between urban and rural areas. Urban areas consistently exhibit lower diversity indices, indicating more uniform fertility behaviors (21). This can be attributed to several factors, including better access to healthcare facilities, higher levels of female education, and greater awareness of family planning methods. Studies have shown that urban women tend to have lower fertility rates due to increased economic opportunities, delayed marriages, and greater control over reproductive health (22). In rural areas, however, the diversity index is higher, suggesting that fertility behaviors are more varied and less predictable. Rural women often face barriers such as limited access to family planning services, lower literacy levels, and strong social pressures to bear children (23). Rural areas also have higher levels of poverty, which is associated with higher fertility rates and less access to reproductive health resources (24). The persistence of higher fertility diversity in rural areas emphasizes the need for targeted interventions aimed at improving education, healthcare access, and family planning services in these regions.

This study is subject to certain limitations. The analysis is based on aggregate NFHS data and does not account for individual-level determinants of fertility behavior. The Diversity Index measures the distribution of fertility across age groups but does not explain the underlying causes of fertility variation. Some state–residence combinations were unavailable in specific NFHS rounds, limiting complete comparisons. In addition, the analysis is descriptive in nature and does not include statistical significance testing. Therefore, the findings should be interpreted as indicative of fertility diversity patterns rather than causal relationships.

## Conclusion

The diversity index of age-specific fertility rates provides a comprehensive tool for understanding fertility patterns across different regions and demographic groups in India. The findings from this study demonstrate significant regional and urban-rural disparities in fertility diversity, with states in the south and west showing more uniform fertility patterns, while states in the north and east continue to exhibit high fertility diversity. These variations are shaped by socio-economic, cultural, and demographic factors, and the findings underscore the need for targeted, region-specific family planning and reproductive health policies. Further research is needed to explore the causal relationships between socio-economic development, education, and fertility transitions, and to develop effective interventions that can reduce fertility disparities across India.

## Data Availability

This study was based on a large dataset that is publicly available on DHS website (https://dhsprogram.com/data/) conducted by the MOHFW and International Institute for Population Sciences (IIPS) in India with ethical standards being complied with including informed consent obtained from participants.
